# Defining the fine structure of promoter activity on a genome-wide scale with CISSECTOR

**DOI:** 10.1093/nar/gkad232

**Published:** 2023-04-04

**Authors:** Vincent D FitzPatrick, Christ Leemans, Joris van Arensbergen, Bas van Steensel, Harmen J Bussemaker

**Affiliations:** Department of Biological Sciences, Columbia University, New York, NY, USA; Department of Systems Biology, Columbia University Medical Center, New York, NY, USA; Division of Gene Regulation, Oncode Institute, Netherlands Cancer Institute, Amsterdam, The Netherlands; Division of Gene Regulation, Oncode Institute, Netherlands Cancer Institute, Amsterdam, The Netherlands; Division of Gene Regulation, Oncode Institute, Netherlands Cancer Institute, Amsterdam, The Netherlands; Department of Cell Biology, Erasmus University Medical Center, Rotterdam, The Netherlands; Department of Biological Sciences, Columbia University, New York, NY, USA; Department of Systems Biology, Columbia University Medical Center, New York, NY, USA

## Abstract

Classic promoter mutagenesis strategies can be used to study how proximal promoter regions regulate the expression of particular genes of interest. This is a laborious process, in which the smallest sub-region of the promoter still capable of recapitulating expression in an ectopic setting is first identified, followed by targeted mutation of putative transcription factor binding sites. Massively parallel reporter assays such as survey of regulatory elements (SuRE) provide an alternative way to study millions of promoter fragments in parallel. Here we show how a generalized linear model (GLM) can be used to transform genome-scale SuRE data into a high-resolution genomic track that quantifies the contribution of local sequence to promoter activity. This coefficient track helps identify regulatory elements and can be used to predict promoter activity of any sub-region in the genome. It thus allows *in silico* dissection of any promoter in the human genome to be performed. We developed a web application, available at cissector.nki.nl, that lets researchers easily perform this analysis as a starting point for their research into any promoter of interest.

## INTRODUCTION

The level at which each gene in the human genome is expressed is dictated by a complex interplay between transcription factors and gene-specific regulatory elements encoded in the DNA. Since almost every gene requires a distinct pattern of expression across cell types, the architecture of both the promoter region proximal to the transcription start site (TSS) and the more distal enhancer regions varies widely in terms of the identity, strength, and spatial arrangement of the transcription factor (TF) binding sites they contain (1–3).

Although enhancers are undoubtedly important to control the expression of most genes, it has been estimated that nearly 50% of the variance in genome-wide gene expression in a given cell type may be explained by proximal promoter sequences (4). Thus, it is important to identify and characterize these sequences for each promoter. Towards this goal, one widely used strategy has been to isolate the candidate proximal promoter region and test its ability to drive the expression of a transcriptional reporter in an ectopic setting. Subsequent assays on various sub-regions then serve to identify the smallest part of the promoter that is still capable of (partially) recapitulating the endogenous expression pattern of the gene (5–8). This classic deletion approach, however, is laborious and therefore only feasible for testing a limited set of regions.

More recently, massively parallel reporter assays (MPRAs) were introduced that can test large numbers of reporter constructs simultaneously (9,10). MPRAs have now been extensively used to map promoter or enhancer activity in genomes across a wide range of species and cell types (11–15). In MPRAs designed to detect promoter activity, candidate sequences are cloned in front of a promoter-less transcription unit; hence, they will only produce a transcript if they harbor autonomous promoter activity. A particularly powerful version of this latter assay is SuRE, a MPRA that employs molecular barcoding to query hundreds of millions of random genomic DNA fragments (4,16). While the resulting data provide detailed maps of autonomous promoter activity throughout the genome, it has remained challenging to extract from these data the key sequences that drive the activity of each individual promoter.

We previously reported proof-of-principle (4) that generalized linear modeling (GLM) can be used to deconvolve SuRE data, thereby quantifying how short sequences within each promoter contribute to its overall activity. However, in this previous study we calculated GLM coefficient tracks for only a handful of individual human promoters, and the limited depth of the SuRE data did not allow for high-resolution analysis (4). Here, we further optimized and applied the algorithm to generate full-genome GLM coefficient tracks. As input, we used state-of-the-art SuRE data from two cell types (K562, a leukemia cell line; and HepG2, a liver carcinoma cell line), each with a complexity of nearly 2.4 billion assayed fragments of roughly 150–500 bp, yielding an average genomic coverage of ∼240x (16). This high coverage greatly benefits the resolution of the predictions. Furthermore, we developed an interactive web application (https://cissector.nki.nl) that enables researchers to inspect these profiles, and to view predictions of intrinsic promoter activity for any region in the human genome. This visualization can be used to easily identify the minimal promoter region that retains most of the full promoter activity. Our tools provide a rational starting point for detailed functional dissection of individual promoters.

## MATERIALS AND METHODS

### SuRE datasets

Genome-wide SuRE experimental data were taken from (16), to which we also refer for a description of quality control procedures used. We combined data from all eight SuRE libraries, two libraries constructed from each of four distinct individual human genomes, and each containing ∼300 million barcoded DNA elements. An element is defined as a unique genomic segment, in terms of chromosome, start and end position, and orientation. After processing, each element had associated with it the following set of DNA barcode counts: (i) the number of observations in the sequenced inverted PCR (iPCR) input library, (ii) in the cDNA library for three K562 biological replicates and (iii) in the cDNA library for two HepG2 biological replicates. Data from the eight libraries were collated as described below and then partitioned into genomic blocks of approximately 10 Mb in length. GLM fits were performed independently for each block. Blocks on the same chromosome were chosen so as to be separated by gaps in joint library coverage in order to ensure that no elements belonged to more than one such block.

For validation of our GLM models, a second SuRE dataset (GEO accession GSE206935) was generated in K562 cells from a focused SuRE library consisting of fragments from four bacterial artificial chromosomes (BACs; CTD-3252A18, CTD-3156P24, CTD-2153L18, and CTD-3075C4) containing four genomic regions covering several housekeeping genes (chr5:139961667–140166117, chr1:109571381–109687521, chr6:26115655–26242415, and chr1:155087497–155235687, respectively). Mapping errors cause a small number of BAC fragments to map to positions outside the original BAC regions; these elements were excluded from our analysis.

### GLM analysis of SuRE data

We used a generalized linear model (GLM) based on the Poisson family, with a logarithmic link function guaranteeing that the expected value of the count of each element is positive, which is well-suited for modeling sequencing counts in a regime where the expected counts are low. Note that for the Poisson distribution the expected variance in the count for each element is equal to the expected count itself, i.e. all variation is due to the sampling error associated with the discrete count distribution, as opposed to other sources of variation.

The GLM framework of CISSECTOR allows for the inclusion of both spatial and non-spatial covariates. Non-spatial covariates can include any other information about individual elements that may influence the observed counts. For the GLM used in this paper, the model took the following form:


$$\begin{equation*}{y}_i\ \sim\ {\rm{Poisson}}\left( {{e}^{{\mu }_i}} \right)\end{equation*}$$

where


$$\begin{equation*}{\mu }_i = \mathop \sum \limits_{j = 1}^J {x}_{ij}{\beta }_j + L_i{\theta }_L + \mathop \sum \limits_{k=1}^K {z}_{ik}\left( {\theta _{k,0} + \left( {\log {n}_i} \right) \theta_{k,1} + {{\left( {\log {n}_i} \right)}}^{2\ }\theta _{k,2}} \right)\end{equation*}$$

Here, ${y}_i$ represents the barcode count for element $i$, which is modeled as a Poisson-distributed variable with mean ${e}^{{\mu }_i}$. For each cell line (K562 or HepG2), barcode counts from biological replicates within each library were summed, yielding a single count ${y}_i$ for each cell type. Since these counts have not been normalized for input or sequencing depth, the pooling step effectively gives each biological replicate leverage over the model coefficient estimation proportional to its sequencing depth. The coefficient$\ {\beta }_j$, corresponding to genomic bin *j*, contributes to the predicted mean of element $i$ only if $x_{ij}\in[0,1]$, which indicates whether element $i$ overlaps with genomic bin $j$, is non-zero. Since a logarithmic link function was used, coefficients reflect the estimated additive effect of the corresponding covariate on the natural logarithm of the expected count. The coefficient estimated for each bin reflects the orientation-specific effect of including it in the genomic segment that drives the reporter. Bins for a given orientation are non-overlapping and collectively cover all genomic regions with non-zero SuRE library coverage.

Theoretically, at a high enough library complexity, the number of spatial covariates for a given strand could approach or equal the combined length of the areas with non-zero coverage, with a uniform bin length of one base-pair and ${x}_{ij}$ set to 1 for all overlapping elements (and 0 for all others). In practice, however, adjacent genomic positions frequently overlap identical sets of elements, leading to redundancy among predictors and convergence issues due to collinearity. To address this technical issue, we chose to group each set of consecutive genomic positions that overlap the same set of elements into a genomic ‘bin’. A single coefficient ${\beta }_j$ was fit for that bin. To facilitate implementation of penalized regression (see below), the indicator ${x}_{ij}$ was set to $\sqrt{\ell_j}$, the square root of the length ($\ell_j$) of bin *j*, for all elements *i* that contain bin *j*, and to zero otherwise. Finally, for the purpose of making predictions outside the training set, the predicted effect of including any additional single genomic base pair in the reporter construct was taken as ${\beta }_j$ divided by $\sqrt{\ell_j}$.

Various non-spatial covariates can be included in a CISSECTOR fit. The ones used in this paper reflect the particular structure of the SuRE dataset. ${\theta }_L$ captures the effect of variation in element length ($L_i$) on SuRE element expression, presumably due to differences in transfection efficiency for plasmids of different length. Data for each of the $K\ = \ 8$ distinct SuRE libraries used in the combined model were generated separately, resulting in library-specific differences in sequencing depth for both input (iPCR) and cDNA barcode counts. In the equation above, $z_{ik}\in[0,1]$ is an indicator variable that is set to unity if element $i$ is from library $k$, and otherwise to zero. $\theta_{k,0}$ is a library-specific coefficient that primarily accounts for differences in the combined sequencing depth of the biological replicates for library $k$. We originally expected input counts to have a simple linear relationship with expression counts, given that they reflect input library element concentrations. However, we observed weakly non-linear and library-specific relationships between input count and mean barcode count (Supplemental Figure S1), possibly as a consequence of low per-element input sequencing depth. To better capture these non-linear relationships, the natural logarithm of the input count ${n}_i$ and the square of that logarithm were both included as a covariate in the model, and library-specific coefficients $\theta_{k,1}$ and $\theta_{k,2}$ estimated for each.

### Penalization

Poisson regression fits were implemented using the R package *glmnet* (17). Coefficients of spatial covariates were penalized to avoid overfitting and address the strong natural collinearity of adjacent genomic bins. This was done using elastic net (18) which imposes penalties on both the L_1_ and L_2_ norm of the coefficients. Both penalties discourage overfitting. Additionally, the L_1_ penalty (${\lambda }_1$) promotes sparsity (i.e. coefficients for inactive genomic regions are more likely to be set to zero) while the L_2_ penalty (${\lambda }_2$) helps address collinearity by encouraging similar coefficient estimates for neighboring bins that share many SuRE elements. Functions in *glmnet* make use of an alternative parameterization, $\alpha$ and $\lambda$, such that ${\lambda }_1 = \alpha \lambda$ and ${\lambda }_2 = ( {1 - \alpha } )\lambda$. To preserve sparsity of the design matrix, no standardization of covariates was used. Instead, the value of the covariate for all elements overlapping a given bin was set to the square root of the length of the bin. This is motivated by the Bayesian conception of penalty parameters in terms of a prior distribution for coefficients (19). As all coefficients share the same penalty parameters, they share the same prior mean (equal to zero) and variance. If a bin has a length L, setting the covariate value for overlapping elements to L^1/2^ ensures that the prior variance is the same regardless of whether each base pair is modeled separately or as part of a single block.

### Tuning of penalty parameters

To reduce computational costs, we partitioned the dataset into continuous segments of overlapping library fragments, separated by regions of zero coverage. Consecutive segments were next combined into 361 genomic blocks of up to 8Mbp each. This length allows for different segments of the genome to be fit efficiently in parallel, circumventing memory limitations that did not allow for a single simultaneous genome-wide fit. The glmnet function was used on a single genomic block to fit the optimal penalty parameters for the data from each cell type. Given an $\alpha$penalty parameter value, glmnet (17) efficiently fits a series of models, each with a different $\lambda$ penalty parameter value. To validate the $\lambda$ parameter for a given $\alpha$, an optimal $\lambda$ value was chosen based on the model that maximized the log-likelihood of a test dataset (a random sample consisting of 10% of all blocks). The initial, largest $\lambda$ in the ‘$\lambda$ path’ was selected to ensure that all coefficients in this model are penalized to zero, with the rest of the $\lambda$ path being a decreasing series evenly spaced in log-space. If the optimal $\lambda$ selected via this method was also the smallest $\lambda$ tested, a series of even smaller $\lambda$s was tested. In all cases, the smallest $\lambda$ eventually produced an inferior model, and the optimal $\lambda$ was selected. This $\lambda$ selection procedure was repeated at various values of $\alpha$, and the optimal $\alpha$ value was based on the $( {\alpha ,\lambda } )$ pair that maximized the test log-likelihood. Optimal penalty parameters were similar but not identical for the K562 and HEPG2 genome-wide fits, with $\alpha$ values of 4 × 10^−3^ and 3 × 10^−3^, and log($\lambda$) values of 7.8 and 8.2, respectively.

### Strategy for performing genome-wide fits

After determining the optimal penalty parameters for each cell type, to reduce computational complexity, we calculated coefficients in a two-step process. First, non-spatial coefficients were estimated in parallel across all 361 distinct genomic blocks. The average of these coefficients was used to produce genome-wide estimates for the effects of these unpenalized non-spatial parameters. These coefficients were then used to calculate ‘offsets’ for each element, effectively fixing the effect of the non-spatial coefficients on expression across all subsequent fits genome-wide. In the second step, these offsets were used to estimate spatial covariates. In each step, genomic blocks were modeled in parallel, which greatly reduced the time required to run the model. For each cell type, a coefficient track with single base-pair coefficients was constructed from the predicted coefficients of the variable-width bins by dividing the bin coefficients by the square root of the width. These coefficients were assigned to every base-pair within the variable-width bin.

### Expression prediction

Expression for a specific hypothetical fragment was predicted using the coefficient track. For the region under consideration, a corresponding vector of single-base-pair coefficients was imported. The length parameter of the model was added to every coefficient to account for this non-spatial covariate. Expression associated with any particular genomics window was predicted by exponentiating of the sum of these rescaled single-base-pair coefficients. Since the other non-spatial covariates were either library-specific or fragment-specific, they were ignored. As a result, predicted expression levels can be interpreted as the mean expected expression count after normalizing for input concentration in the library and accounting for sequencing depth.

### Validation using BAC library

SuRE data generated using the BAC library in K562 cells were used to test the performance of our GLM predictions on unobserved fragments. BAC DNA fragments with start and end positions that were identical to that of any fragment in one of the genome-wide SuRE libraries were removed prior to analysis. For each BAC element starting and ending within 1kb of an annotated transcription start site, predictions were generated using the K562 genome-wide GLM coefficient track. These predictions included the length penalty from the original model, but did not include any other unpenalized coefficients, which correspond to specific libraries used in the original model. Because sequencing depth can influence the magnitude of cDNA barcode counts in a SuRE experiment, these predictions are expected to be proportional to, but not equal to, the observed expression counts. While the per-element sequencing depth of K562 BAC SuRE experiments was higher than for genome-wide K562 ones, the effects of dropout and general overdispersion nevertheless resulted in relatively high-variance observed counts. To address this, BAC DNA fragments were first grouped together based on similar endpoints. Specifically, each TSS region was split into 50 bp bins, and each element within a TSS region was assigned to a start bin and an end bin. Elements with the same start and end bin were grouped together. The mean log predicted expression and the log of the mean observed expression for each group was calculated. All Pearson correlations were calculated in log-space.

### Replicate correlation analysis

To assess inter-cell-type versus intra-cell-type reproducibility, we performed replicate-specific fits on an arbitrarily chosen 8Mbp subset of chr17 (for computational efficiency). Each of the eight SuRE library datasets contained three K562 biological replicates, two HepG2 biological replicates, and one iPCR replicate. One replicate was selected from each library to generate three distinct K562 datasets and two distinct HepG2 datasets, which were then fit separately. Correlations between the resulting tracks were then computed. Because only a single iPCR replicate is available, these fits were based on the same iPCR counts. As these input counts are shared across cell types, they cannot explain the difference between intra-cell-type and inter-cell-type correlations.

### Cross-correlation analysis

We selected TFs for which we had both a motif and ChIP data from ENCODE available. As a source of TF motifs, we used a curated set of position weight matrices from Ref. (20). These were transformed into a pseudo position-specific affinity matrix (pseudo-PSAM) by dividing the nucleotide counts of each position in the motif over the most abundant nucleotide (21). Optimal IDR-thresholded ChIP-seq peaks were downloaded from ENCODE (22,23). These peaks are typically several hundred base-pairs wide, and only indicate the broad region bound by a TF. To better capture the TF affinity landscape within these regions, sequences of every peak were used to calculate total TF affinity scores at each position in the peak by summing over a sliding window (all PSAM positions overlapping a position) as well as both strands. The resulting score was assigned to the central nucleotide position of the pseudo-PSAM. We then ran a cross correlation on 1 kb regions surrounding 22104 well-annotated promoters between these affinity score profiles and the CISSECTOR GLM, and calculated the average for each lag. TF affinity auto-correlation profiles, calculated in the same way, were also calculated to assess how the spatial distribution of affinities might influence cross-correlation.

## RESULTS

### Design of the generalized linear model (GLM) underlying CISSECTOR

**Figure 1.**
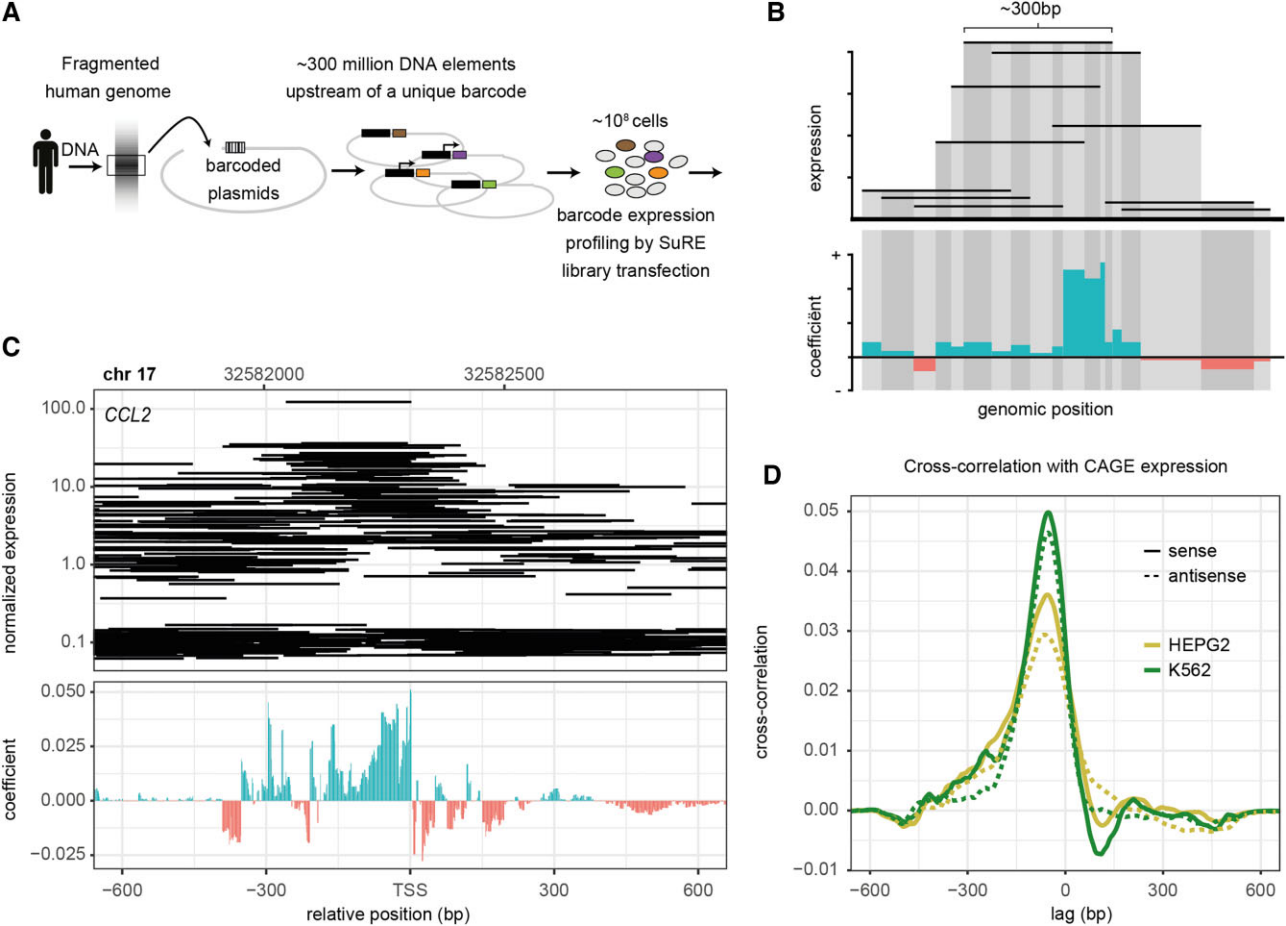
Overview of CISSECTOR GLM methodology. (**A**, **B**) In the SuRE assay, genomes of one or more individuals are fragmented, and random fragments of ∼300bp are cloned into a plasmid upstream of a barcode and a promoter-less reporter. Inverted PCR (iPCR) of the library is used to associate each barcode with its associated genomic fragment; iPCR counts also serve to assess the relative abundance of each barcode in the library. Reporter expression after transfection to a host cell line was assayed by counting barcodes in a cDNA library. For the GLM analysis, the genome was divided into bins of variable size based on an alignment of all fragments in the library. The relative expression level associated with each barcode was modeled in terms of multiplicative contributions from each genomic bin included in the associated genomic fragment. For each bin, a model coefficient was determined using a maximum-likelihood fit of the model. (**C**) Example promoter (*CCL2*) showing iPCR-normalized SuRE cDNA counts. Each horizontal line represents a barcoded fragment from its start to end on the x-axis. Only fragments that had the same orientation in the plasmid as the leading (+) strand of the genome are displayed. Panel underneath shows the coefficient of each genomic bin in the CISSECTOR model. (**D**) Cross-correlation between CISSECTOR coefficients and CAGE expression for 1kb regions surrounding 22104 well-annotated promoters. Lag shows base-pair shift in CAGE expression matrix relative to CISSECTOR coefficients. Correlation was smoothed using a running mean across a 25bp window.

In SuRE (Figure 1A), millions of randomly sheared genomic DNA fragments are cloned into a plasmid, upstream of a generic transcription unit that carries a barcode that is unique for each fragment. After a library sequencing procedure that maps each barcode to the corresponding genomic fragment, cultured cells are transfected with the library. Because the plasmid backbone does not contain a promoter, transcription of the downstream barcode-containing reporter is a direct measure of the promoter activity encoded in the upstream genomic fragment. This is activity is measured by counting barcodes in reporter RNA isolated from the transfected cells. Because of the very large number of random DNA fragments that are assayed in parallel, autonomous transcriptional activity of each promoter is probed for a large number of distinct, partially overlapping fragments (Figure 1B, top panel). Highly-expressed fragments tend to overlap each other (Figure 1C), which suggests that some part of their shared sequence is responsible for driving this expression. However, many of these fragments extend up- and downstream of the segments that are apparently shared by active fragments, into regions that make no obvious contribution to expression. As a consequence, the location of individual highly-expressed fragments provides only a rough indication of which genomic segments are important for local transcriptional regulation.

We implemented a regularized Poisson GLM approach, named CISSECTOR, that dissects the *cis-*regulatory influences on reporter fragment expression by modeling the expected expression count as the product of contributions from every base-pair position contained within a genomic fragment. CISSECTOR builds upon an earlier prototype model (4) that was used to dissect a small number of promoter regions, scaling this approach to the genome-wide scale. It models reporter expression in a given cell type as Poisson-distributed counts, and uses elastic net penalization (17) to avoid over-fitting and address the high degree of collinearity in covariates corresponding to neighboring genomic positions. Penalized covariates correspond to short stretches of the genome (‘bins’) that are overlapped by some number of reporter fragments (Figure 1B, shading). Once fit, the coefficient for each bin is divided equally among its composite genomic positions, resulting in an estimate of per-base-pair contributions to reporter expression activity. To account for the directionality of expression, we fit our model separately for the Watson (forward or +) and Crick (reverse or –) strands of the human genome. Additional unpenalized covariates were included in the model to account for technical biases, such as input library counts, and element length (see Materials and Methods). We also make use of library-specific unpenalized covariates when combining results from multiple libraries for the same cell type.

Aside from the difference in scale, our model differs in a number of ways from the earlier prototype (4). Previously, fixed-width genomic bins were used, resulting in only partial overlap between a bin and any reporter fragments that either started or ended within that bin. This can result in the expression of reporter fragments influencing the estimated activity of nearby genomic positions with which they do not directly overlap. These artifacts could be avoided by using a covariate for every single genomic base-pair, but fitting such a model is extremely inefficient. To identify a smaller set of genomic bins that avoid the partial overlap issue, CISSECTOR takes advantage of the fact that the start- and end-points of all fragments in a given MPRA library only correspond to a subset of all possible positions. Rather than using fixed-width bins, CISSECTOR allows variable-width bins, with each bin corresponding to the set of neighboring positions that are overlapped by an identical set of reporter fragments (Figure 1B). The resulting bin lengths can range in size from 1 bp to the length of whole reporter fragments. In the SuRE datasets used below, 99% of genomic bins were 10bp or shorter, with a median bin length of 2.

The output of the model fit is a genome wide coefficient track that allows the *cis-*regulatory structure of each promoter region to be dissected and interpreted in a biologically intuitive manner (Figure 1B, bottom panel): a positive coefficient for a given bin indicates that the associated DNA sequence has an activating effect on the expression of the promoter, while a negative coefficient indicates that it has a repressive effect.

### Fine-grained prediction of sequences that contribute to promoter activity

Figure 1C shows an example of raw SuRE data and the derived GLM coefficient track from K562 cells, around the TSS of the *CCL2* gene, which encodes a chemokine that is important in immune regulation (24). As might be expected, the coefficient track indicates that activating sequences are concentrated just upstream of the TSS, in this case up to position –352 bp. In addition, two elements around positions –220 and –380 are predicted to harbor repressive activity. Thus, the GLM modeling clearly delineates the extent of the promoter region, and additionally identifies putative activating and repressive elements within it. Some repressive elements and a few weakly activating sequences are also predicted downstream of the TSS, but these should be interpreted with caution as they may reflect the presence of elements that affect RNA stability rather than promoter activity (4). Overall, this example illustrates how CISSECTOR generates a fine-grained prediction of the DNA elements that may contribute to promoter activity, inferred from the SuRE signals obtained with much larger DNA fragments. Additional examples of coefficient tracks will be discussed below.

To show how the GLM coefficient profile relates to transcription initiation we cross-correlated the coefficient tracks with transcription initiation measured by CAGE (Figure 1D). We ran a cross correlation on 1kb regions surrounding 22104 well-annotated promoters. The correlation function peaks around position –50, indicating that the highest concentration of proximal drivers of promoter activity can be found around 50 bp upstream of sites of transcriptional initiation, with cross-correlation in excess of 0.01 in the region between approximately –200 bp and + 50 bp. This is comparable to previously reported GLM implementation on the SuRE library presented in van Arensbergen *et al.* (4).We also correlated the anti-sense GLM coefficient profile to the same CAGE signal which shows that the same elements around promoter regions are also capable of driving anti-sense transcription, be it to a lesser degree.

**Figure 2.**
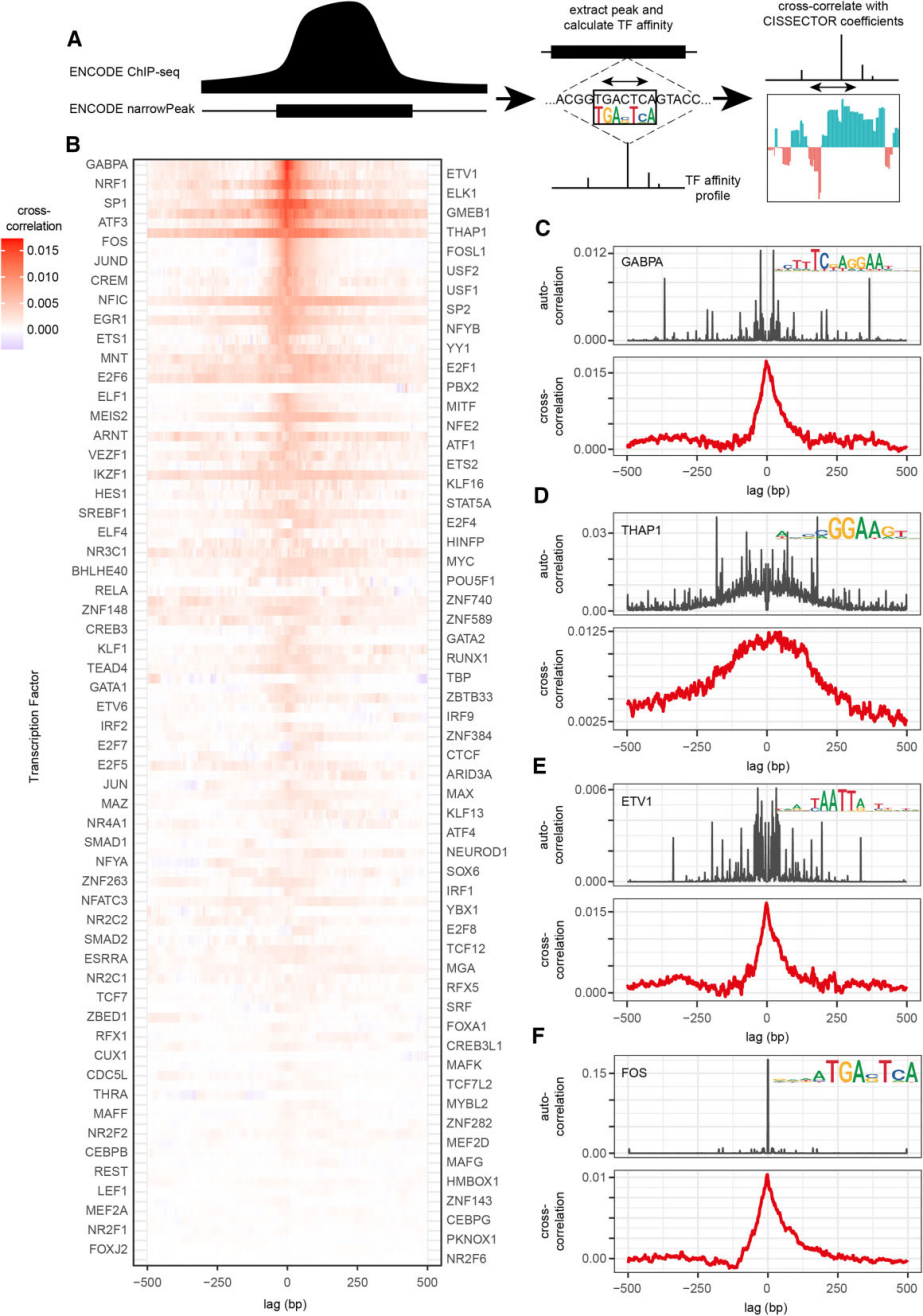
GLM coefficient tracks correlate with TF binding patterns. (**A**) Cartoon representation of comparative analysis between TF-binding and CISSECTOR GLM coefficients. Briefly: binding affinity was scanned across the subset of the genome covered by peaks identified in ChIP-seq data from the ENCODE project for the same TF. Subsequently cross-correlation was performed between affinities and CISSECTOR coefficients focusing on regions 1kb around annotated promoters. (**B**) Average cross-correlation between TF binding and GLM coefficient tracks (averaged over the Watson and Crick strand), across the subset of the genome covered by peaks identified in ChIP-seq data from the ENCODE project for the same TF. (**C–F**) Average cross-correlation plots (bottom panels) between GLM coefficients and the binding of selected TFs. For reference, the average autocorrelation plots for the TF affinity profiles are shown in the top panels. Binding motifs of the TFs are depicted as logos. Lag of 0 was removed from the auto-correlation.

We observed that the CISSECTOR coefficient tracks of individual promoters often include narrow peaks and troughs (Figure 1C, bottom panel; see also examples below), suggesting that the locations of functionally important sequences can be delineated with high genomic resolution. To gain confidence that these detailed features are indeed biologically meaningful, we compared GLM coefficient tracks with the predicted binding of TFs. Specifically, we combined K562 ChIP-seq data (22,23) with TF binding motif models (20,21) to generate affinity profiles within known ChIP-seq peaks for a given TF, then calculated the cross-correlation between these affinity profiles and our K562 coefficient track on the 1 kb regions surrounding 22104 well-annotated promoters (Figure 2A). If narrow peaks in the GLM coefficient track generally coincide with TF binding sites, then the cross-correlation function should be a narrow peak centered at lag = 0. Indeed, this is the case for several TFs (cf. Figure 2B, C, E, F). Other TFs, however, show broader cross-correlation profiles (cf. Figure 2B, D). A plausible explanation for this ‘blurring’ was that the binding sites for these TFs tend to spatially cluster amongst themselves within promoter regions. Therefore, we also computed the auto-correlation functions for the binding affinity distribution of each TF, which may be expected to be broader if binding sites for the same TF tend to be spatially clustered, and narrow if individual binding sites occur independently. Indeed, we observe a broad TF binding affinity auto-correlation profile whenever the cross-correlation between the binding affinity and CISSECTOR coefficient showed a broad peak (Figure 2D). We conclude that the fine-structure of our coefficient GLM profiles reflects, at least in part, effects of local TF binding.

### Using GLM coefficient tracks to define optimal promoter fragments

**Figure 3.**
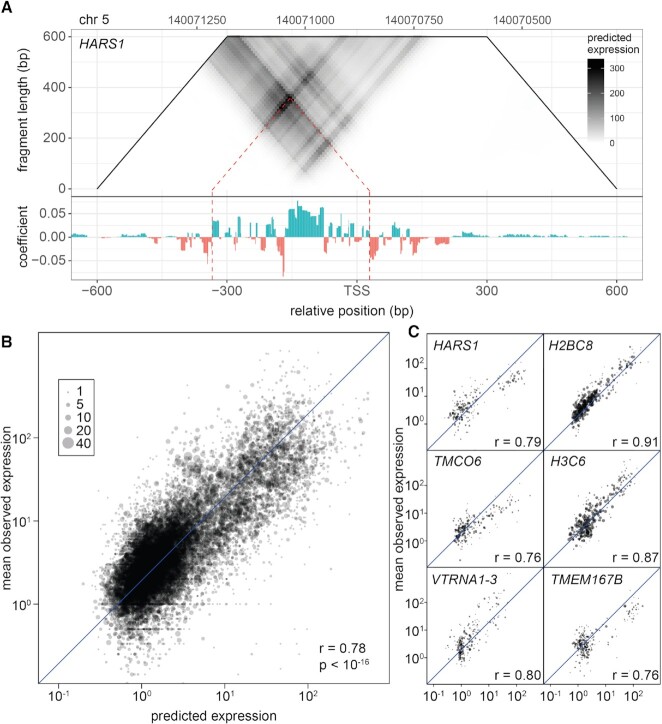
Predicting optimal promoter fragments from GLM coefficient tracks. (**A**) The trapezoid on top shows the prediction for each possible promoter fragment, up to 600bp in size, around the TSS of *HARS1*, as derived from the GLM coefficient track at the bottom. Each fragment is represented by its center point along the x-axis and its size on the y-axis. The red dotted line shows the fragment that is predicted to give rise to the highest reporter expression level. (**B**, **C**) SuRE analysis was performed on a BAC library representing a small subset of the human genome (including *HARS1*), allowing for much higher coverage of the genes contained in the BACs than with the genome-wide library. To further reduce sampling noise, reads were grouped in terms of fragments starting and ending in the same respective 25 bp bin. cDNA counts were normalized by iPCR counts, log-transformed, and then averaged within each such group of fragments. These values were compared to the predictions made by the genome-wide GLM model, either across all genes represented in the BACs (B) or for each gene/TSS separately (C).

The GLM coefficient at any given genomic position represents the predicted change in log-expression if this position were to be included in the reporter construct. By summing these coefficients over a larger range, the autonomous promoter expression for any hypothetical fragment in the human genome can be predicted, even if this fragment was not included in the original SuRE data. Making such predictions for many start/end combinations yields a two-dimensional (2D) map of hypothetical reporter fragment expression. This is illustrated in Figure 3A, which shows the predicted expression for every hypothetical fragment up to 600 bp in a region 600 bp around the TSS of the gene *HARS1*, which encodes a histidyl-tRNA synthetase. We limited this analysis to fragments up to 600 bp in length, because the fragments assayed by SuRE were not larger than this size. The *HARS1* promoter contains a specific subregion from approximately –335 to +30 bp that is predicted to optimally drive expression (Figure 3A, dashed lines). Thus, our 2D visualization of predicted fragment activities facilitates the identification of promoter subregions that may be expected to give the highest promoter activity.

### Experimental validation of predicted fragment activities

To further validate the predictive capability of our model, we set out to experimentally measure the activity of a large number of fragments that were not present in the original SuRE dataset. For this we used a separate, focused SuRE library that consisted of 680 thousand fragments selected from nine genomic loci, together covering 1.3 Mb and including 47 annotated promoters (4). This library is of much lower complexity than the full-genome libraries, resulting in much more precise quantification of relative reporter expression for individual fragments. We compared the predictions made by our genome-wide GLM model to these measured reporter activities, restricting the analysis to fragments that were not directly measured in the genome-wide SuRE data (and hence only predicted). This resulted in an overall Pearson correlation of 0.78 (*P* < 10^−16^) between measured and predicted activities (Figure 3B). This agreement was consistent when fragments belonging to each individual promoter were considered separately (Figure 3C mean Pearson correlation across 47 promoters: 0.76). We conclude that, in general, the GLM model reliably predicts the transcriptional activity of genomic fragments up to 600 bp, even if they were not directly assayed by SuRE.

### Promoter architectures are diverse and cell-type dependent

**Figure 4.**
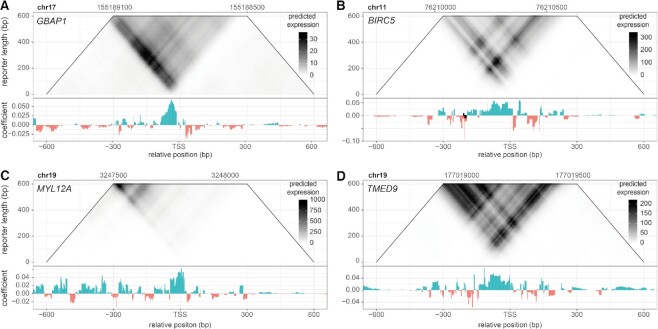
Gallery of GLM coefficient tracks, showing the rich variation from gene to gene. The same representation is used as in Figure 3. Gene names are indicated at the top left of each panel.

Our genome-wide coefficient tracks and 2D visualization of expression across all possible promoter fragments can be used to explore the diversity of promoter architectures in the human genome. Examples are shown in Figure 4, illustrating a rich variety in predicted regulatory element distribution from promoter to promoter. Some promoters have a very large extent (e.g. *MYL12A*, >600 bp), while others encode almost all their activity in the first 100 bp upstream (e.g. *GBAP1*). For other promoters, such as *BIRC5*, while a specific fragment of the promoter is predicted to maximize expression, either extending or reducing it is expected to reduce activity.

We also investigated differences in the coefficient profiles between K562 and HepG2 cells. While fit separately, the SuRE data for these two cell types were generated with exactly the same fragment libraries, and therefore the coefficient tracks could be compared directly. The modest genome-wide correlation between the respective coefficient tracks across both strands (*r* = 0.39) suggests that there are substantial differences between the cell lines. To confirm that this correlation reflected cell type-specific differences, we split the dataset for each cell type by biological replicates (three in K562, two in HepG2) and generated separate fits with each replicate for the same subset of the genome. Correlations between replicates from the same cell type (mean *r* = 0.60) were consistently and significantly (*P* = 0.01, *t*-test) higher than correlations between replicates from different cell types (mean *r* = 0.39); for details, see Supplemental Figure S2). Although strictly speaking our analysis could still be confounded by cell-type-specific differences in technological bias associated with the reporter assay, this result nevertheless suggests that CISSECTOR profiles capture cell-type specific differences in regulatory activity.

**Figure 5.**
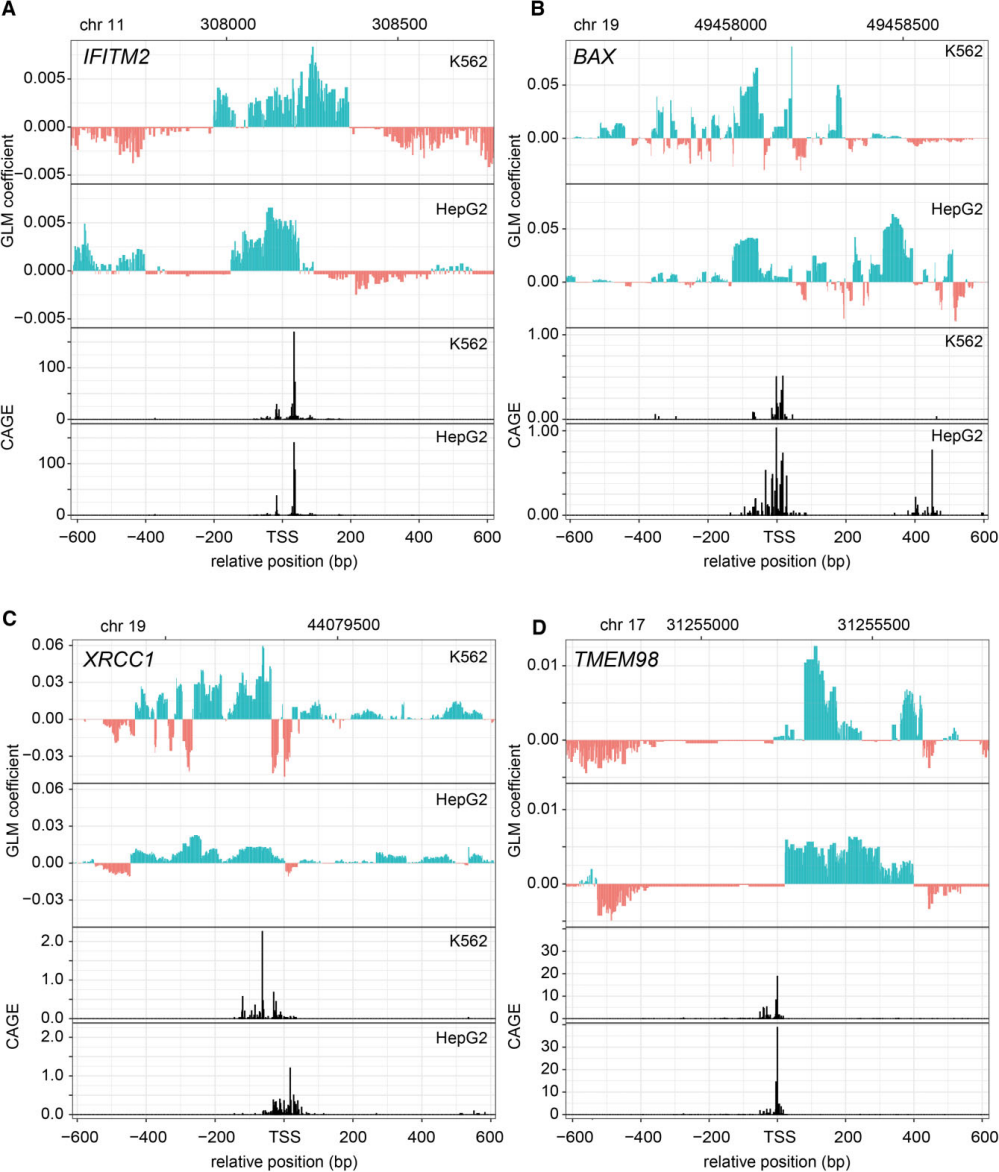
GLM coefficient tracks provide information about cell-type-specific gene regulation. For each gene/TSS, the two upper panels show the GLM coefficients tracks in the sense direction for K562 and HepG2 cells, respectively. The two lower panels show endogenous transcriptional activity as assayed using CAGE, also in the sense orientation. Gene names are indicated at the top left of each panel.

We also observed striking differences in the coefficient tracks for some individual promoters that are active in both cell types. For example, *IFITM2* has the same TSS in K562 and HepG2 according to published CAGE data (25), yet the upstream GLM coefficient profile suggests that partially distinct sequence elements drive promoter activity in each context (Figure 5A). Another striking example is the *XRCC1* promoter (Figure 5C). CAGE data for this promoter show that transcriptional initiation happens about 100 bp further upstream in HepG2 than in K562 cells. Remarkably, the GLM coefficient profiles show two K562-specific patches of strongly negative values that coincide with the TSS position in HepG2 cells. This strongly suggests that two short repressive elements repress transcription initiation at this position in K562 cells, but not in HepG2 cells. Interestingly, for the *BAX* gene, CAGE data indicate that a secondary promoter 400–450 bp downstream of the TSS is only active in HepG2 cells (Figure 5B). The GLM profile indicates that this is likely attributable to a stretch of ∼100 bp of sequence immediately upstream of this secondary promoter, possibly assisted by additional sequence elements that show positive coefficients in HepG2 cells only. Finally, we found TMEM98 to have yet another striking configuration, in which for both cell lines the main stretch of sequence predicted to be responsible for driving expression, lays downstream, rather than upstream of the TSS.

### A web tool for viewing promoter GLM data

Together, the examples above illustrate how the GLM coefficient tracks and the derived 2D visualization can help researchers to understand the architecture of individual promoters, to identify putative activating and repressive elements, and to delineate the region of a promoter that is likely to harbor most of its regulatory sequences. To provide easy access to these data, we implemented a simple web interface that is freely accessible at https://cissector.nki.nl. Users can visualize the coefficient tracks and 2D plots by entering a gene name, ENSEMBL ID, or genomic coordinates. An automatic link to the UCSC Genome Browser is provided for further integration with other genome annotation data. Interactive inspection of the 2D and coefficient tracks facilitates the identification of promoter subregions of interest. Furthermore, promoter activities of sets of genomic fragments can be calculated in batch format.

## DISCUSSION

Through GLM-based analysis of SuRE data we have derived fine-grained genome-wide coefficient tracks that can be used to analyze and predict promoter activity across the human genome. These coefficient tracks and the derived 2D plots highlight the subregions of each promoter that are most likely to be relevant for its functioning, and provide clues about what the effect might be of introducing mutations at a specific location. Our data-driven *in silico* promoter dissection can help focus wet-lab experimental efforts and generate new hypotheses for any promoter of interest. In addition, our method is able to predict expression for any hypothetical promoter fragment (up to a reasonable size, see below).

CISSECTOR improves interpretability of SuRE data and helps reduce intrinsic noise due to low read counts for individual unique SuRE fragments. At any given locus, SuRE data will include reporter counts for many overlapping fragments. This makes them a natural source of information for predicting the boundaries and activity of promoter-associated regulatory elements. However, we had to address some technical challenges associated with extracting accurate predictions from SuRE results: High-expression SuRE elements must contain functional promoter regions, but may be accompanied by flanking non-functional regions which could be removed without reducing overall expression. The expression of individual elements can also be highly variable due to transfection-related drop-out, natural cell heterogeneity, and other sources of experimental noise, making single-element counts fairly inaccurate predictors of reporter expression. Additionally, even high-coverage SuRE libraries cannot provide an exhaustive survey of all possible start/end combinations at a given locus, making it difficult to predict the expression of unobserved elements. CISSECTOR overcomes these challenges by summarizing information from all observed elements in terms of a single genome-wide predictive coefficient track.

Although with some examples we tried to explain cell-type-specific differences in the GLM track between K562 and HepG2 cells, we stress that the model we used is purely based on sequence geometry in terms of start and end positions of fragments. It does not explicitly consider the base sequence of the DNA, and as such, its purpose is solely to locally deconvolve the SuRE data so as to achieve precision on a spatial scale much smaller than the size of a typical SuRE fragment or the width of a typical SuRE coverage peak (Figure 1). The main benefit of our GLM tracks is that they are easily interpretable and allow one to deal with varying fragment sizes, coverage, and data sparsity in a coherent manner.

Approaches that explicitly try to explain functional genomics data such as ChIP-seq, ATAC-seq, DNA-seq or MPRA in terms of the underlying DNA sequence, with the goal of predicting activity for unseen or mutated sequences, such as deep learning algorithms (26–29) address a different question. It will be interesting to see how well they perform on SuRE data, and how similar or different the predictions by the geometry-based and sequence-based methods would be when it comes to defining optimal promoters.

The current SuRE-based GLM predictions also have limitations. Because the SuRE assay is based on transiently transfected plasmids, not all detected signals necessarily reflect promoter activity in the native genomic context. For example, some promoters are repressed by heterochromatin in their natural context, or substantially dependent on distal enhancers. Nevertheless, the genome-wide correlation of about 0.5 between SuRE activity and endogenous promoter activity (4) suggests that GLM predictions will often be useful. Careful comparison of the GLM predictions with measures of endogenous TSS activity (e.g. CAGE, PRO-cap or GRO-cap (30–32) can help interpret the results. The main value of our GLM predictions is that they can help identify key regions of individual promoters, and generate concrete hypotheses to focus labor-intensive mutagenesis experiments. Furthermore, because the assayed fragments in the used SuRE datasets were generally <500 bp in size (16), only coefficients within this distance from a known TSS are easily interpretable, with transcription initiating from the same start site, when present in the plasmid context. GLM signals further away may point to an ectopic site of transcription initiation in the plasmid context.

In this study we focused on promoters. However, SuRE can also be used to study enhancers, because most enhancers drive low-level expression, and this activity correlates with other measures of enhancer activity (4). Furthermore, we expect that our GLM approach can be applied to data from MPRAs that specifically query enhancer activity, such as STARR-seq (33), as long as the fragment libraries are of sufficient complexity and genome coverage. We anticipate that the number of cell types and species assayed by SuRE and other MPRAs will rapidly increase, offering opportunities to study cell-type and species-specific differences and similarities in promoter and enhancer architectures.

**Figure 6.**
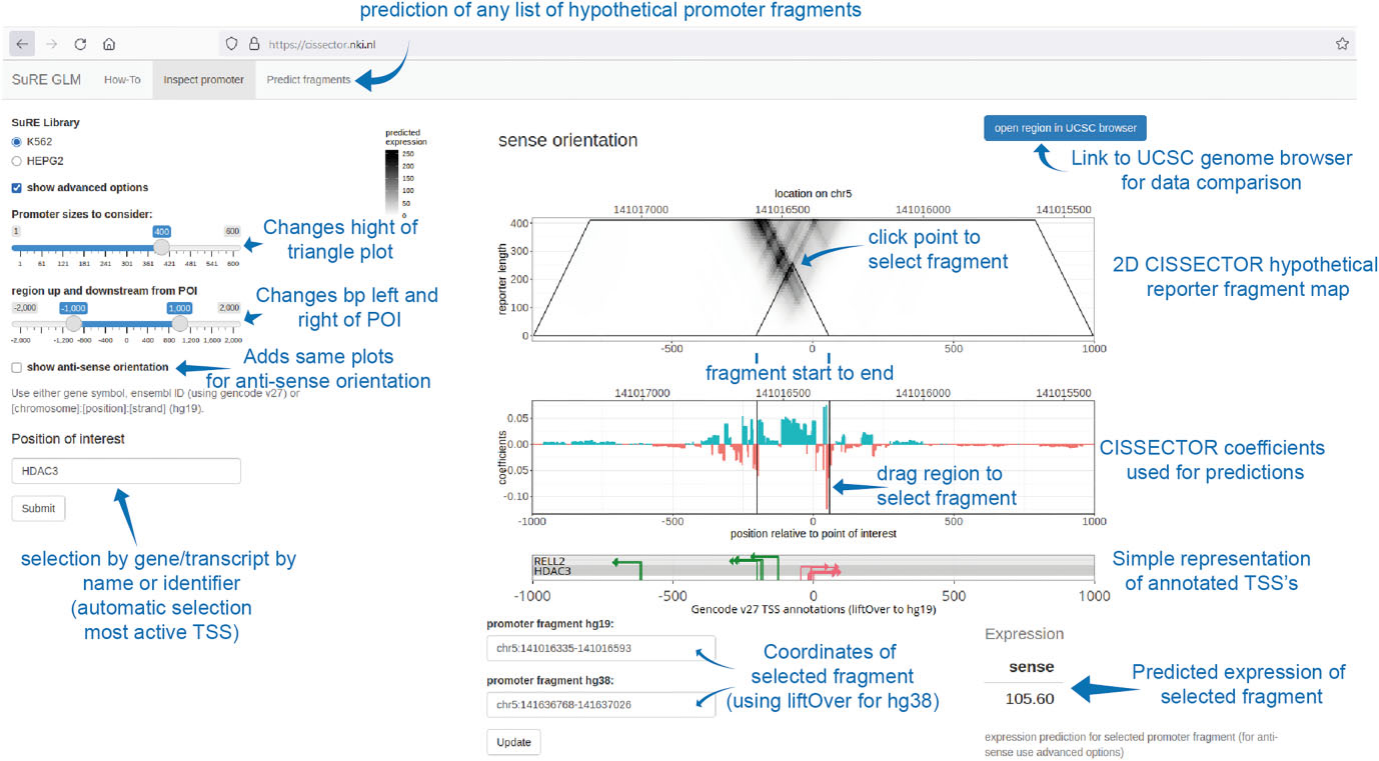
Screenshot of CISSECTOR webtool. Using this webtool, researchers interested in a particular promoter region can easily browse the coefficient tracks and inspect our predictions of transcriptional activity for many different truncations of the same promoter regions. Firstly, a ‘Position of interest’ needs to be selected around which the triangle representation is generated and displayed. Subsequently, by either selecting a point in the triangle, selecting a region in the coefficient track, or by using the ‘Promoter fragment’ text boxes, predictions can be made of a putative reporter construct with the selected sequence. In addition, there is a tab pane that allows the prediction of any list of genomic fragments (accepting BED-format).

We discussed several examples of how our understanding of promoters can be gained by inspecting the CISSECTOR coefficient tracks in the context of other genomic features. However, we are aware that our analyses just skim the surface of what might be learned from our GLM tracks. Expert domain knowledge is required to properly interpret them in the right context. To facilitate other researchers, we have designed a webtool (https://cissector.nki.nl) using which researchers interested in a particular promoter region can easily browse the coefficient tracks and inspect our predictions of transcriptional activity for many different truncations of the same promoter regions (Figure 6).

## DATA AVAILABILITY

The SuRE data that were used for validation are available under GEO accession GSE206935. Our code is available at https://github.com/BussemakerLab/CISSECTOR and https://doi.org/10.5281/zenodo.7750893.

## Supplementary Material

gkad232_Supplemental_File

## Notes

Present address: Joris van Arensbergen, Annogen B.V., Amsterdam, The Netherlands.

## References

[1] Schoenfelder S. , Fraser P. Long-range enhancer-promoter contacts in gene expression control. Nat. Rev. Genet. 2019; 20:437–455. 31086298 10.1038/s41576-019-0128-0

[2] Zabidi M.A. , Arnold C.D., Schernhuber K., Pagani M., Rath M., Frank O., Stark A. Enhancer-core-promoter specificity separates developmental and housekeeping gene regulation. Nature. 2015; 518:556–559. 25517091 10.1038/nature13994 PMC6795551

[3] Ray-Jones H. , Spivakov M. Transcriptional enhancers and their communication with gene promoters. Cell. Mol. Life Sci. 2021; 78:6453–6485. 34414474 10.1007/s00018-021-03903-w PMC8558291

[4] van Arensbergen J. , FitzPatrick V.D., de Haas M., Pagie L., Sluimer J., Bussemaker H.J., van Steensel B. Genome-wide mapping of autonomous promoter activity in human cells. Nat. Biotechnol. 2017; 35:145–153. 28024146 10.1038/nbt.3754 PMC5498152

[5] Fujimaki T. , Huang Z.Y., Kitagawa H., Sakuma H., Murakami A., Kanai A., McLaren M.J., Inana G. Truncation and mutagenesis analysis of the human X-arrestin gene promoter. Gene. 2004; 339:139–147. 15363854 10.1016/j.gene.2004.06.032

[6] Langdon S.D. , Kaufman R.E. Gamma-globin gene promoter elements required for interaction with globin enhancers. Blood. 1998; 91:309–318. 9414299

[7] Hooven L.A. , Vorachek W.R., Bauman A.B., Butler J.A., Ream L.W., Whanger P.D. Deletion analysis of the rodent selenoprotein W promoter. J. Inorg. Biochem. 2005; 99:2007–2012. 16099510 10.1016/j.jinorgbio.2005.06.035

[8] Xu Y.Z. , Kanagaratham C., Jancik S., Radzioch D Promoter deletion analysis using a dual-luciferase reporter system. Methods Mol. Biol. 2013; 977:79–93. 23436355 10.1007/978-1-62703-284-1_7

[9] Patwardhan R.P. , Hiatt J.B., Witten D.M., Kim M.J., Smith R.P., May D., Lee C., Andrie J.M., Lee S.I., Cooper G.M. et al. Massively parallel functional dissection of mammalian enhancers in vivo. Nat. Biotechnol. 2012; 30:265–270. 22371081 10.1038/nbt.2136 PMC3402344

[10] Melnikov A. , Murugan A., Zhang X., Tesileanu T., Wang L., Rogov P., Feizi S., Gnirke A., Callan C.G. Jr, Kinney J.B et al. Systematic dissection and optimization of inducible enhancers in human cells using a massively parallel reporter assay. Nat. Biotechnol. 2012; 30:271–277. 22371084 10.1038/nbt.2137 PMC3297981

[11] Chatterjee S. , Ahituv N. Gene regulatory elements, major drivers of human disease. Annu. Rev. Genomics Hum. Genet. 2017; 18:45–63. 28399667 10.1146/annurev-genom-091416-035537

[12] Santiago-Algarra D. , Dao L.T.M., Pradel L., Espana A., Spicuglia S. Recent advances in high-throughput approaches to dissect enhancer function. F1000Res. 2017; 6:939. 28690838 10.12688/f1000research.11581.1 PMC5482341

[13] Kwon S.B. , Ernst J. Investigating enhancer evolution with massively parallel reporter assays. Genome Biol. 2018; 19:114. 30107810 10.1186/s13059-018-1502-5 PMC6090676

[14] Kinney J.B. , McCandlish D.M. Massively parallel assays and quantitative sequence-function relationships. Annu. Rev. Genomics Hum. Genet. 2019; 20:99–127. 31091417 10.1146/annurev-genom-083118-014845

[15] Trauernicht M. , Martinez-Ara M., van Steensel B. Deciphering gene regulation using massively parallel reporter assays. Trends Biochem. Sci. 2020; 45:90–91. 31727407 10.1016/j.tibs.2019.10.006

[16] van Arensbergen J. , Pagie L., FitzPatrick V.D., de Haas M., Baltissen M.P., Comoglio F., van der Weide R.H., Teunissen H., Vosa U., Franke L. et al. High-throughput identification of human SNPs affecting regulatory element activity. Nat. Genet. 2019; 51:1160–1169. 31253979 10.1038/s41588-019-0455-2 PMC6609452

[17] Friedman J. , Hastie T., Tibshirani R. Regularization paths for generalized linear models via coordinate descent. J. Stat. Softw. 2010; 33:1–22. 20808728 PMC2929880

[18] Zou H. , Hastie T. Regularization and variable selection via the elastic net. J. Roy. Stat. Soc. B (Stat. Methodol.). 2005; 67:301–320.

[19] Li Q. , Lin N. The Bayesian elastic net. Bayesian Anal. 2010; 5:151–170.

[20] Diaferia G.R. , Balestrieri C., Prosperini E., Nicoli P., Spaggiari P., Zerbi A., Natoli G. Dissection of transcriptional and cis-regulatory control of differentiation in human pancreatic cancer. EMBO J. 2016; 35:595–617. 26769127 10.15252/embj.201592404 PMC4801945

[21] Foat B.C. , Tepper R.G., Bussemaker H.J. TransfactomeDB: a resource for exploring the nucleotide sequence specificity and condition-specific regulatory activity of trans-acting factors. Nucleic Acids Res. 2008; 36:D125–D131. 17947326 10.1093/nar/gkm828 PMC2238954

[22] Davis C.A. , Hitz B.C., Sloan C.A., Chan E.T., Davidson J.M., Gabdank I., Hilton J.A., Jain K., Baymuradov U.K., Narayanan A.K. et al. The Encyclopedia of DNA elements (ENCODE): data portal update. Nucleic Acids Res. 2018; 46:D794–D801. 29126249 10.1093/nar/gkx1081 PMC5753278

[23] Encode Project Consortium An integrated encyclopedia of DNA elements in the human genome. Nature. 2012; 489:57–74. 22955616 10.1038/nature11247 PMC3439153

[24] Lim S.Y. , Yuzhalin A.E., Gordon-Weeks A.N., Muschel R.J. Targeting the CCL2-CCR2 signaling axis in cancer metastasis. Oncotarget. 2016; 7:28697–28710. 26885690 10.18632/oncotarget.7376 PMC5053756

[25] Lizio M. , Abugessaisa I., Noguchi S., Kondo A., Hasegawa A., Hon C.C., de Hoon M., Severin J., Oki S., Hayashizaki Y. et al. Update of the FANTOM web resource: expansion to provide additional transcriptome atlases. Nucleic Acids Res. 2019; 47:D752–D758. 30407557 10.1093/nar/gky1099 PMC6323950

[26] Movva R. , Greenside P., Marinov G.K., Nair S., Shrikumar A., Kundaje A. Deciphering regulatory DNA sequences and noncoding genetic variants using neural network models of massively parallel reporter assays. PLoS One. 2019; 14:e0218073. 31206543 10.1371/journal.pone.0218073 PMC6576758

[27] Alipanahi B. , Delong A., Weirauch M.T., Frey B.J. Predicting the sequence specificities of DNA- and RNA-binding proteins by deep learning. Nat. Biotechnol. 2015; 33:831–838. 26213851 10.1038/nbt.3300

[28] de Almeida B.P. , Reiter F., Pagani M., Stark A. DeepSTARR predicts enhancer activity from DNA sequence and enables the de novo design of synthetic enhancers. Nat. Genet. 2022; 54:613–624. 35551305 10.1038/s41588-022-01048-5

[29] Rube H.T. , Rastogi C., Feng S., Kribelbauer J.F., Li A., Becerra B., Melo L.A.N., Do B.V., Li X., Adam H.H. et al. Prediction of protein-ligand binding affinity from sequencing data with interpretable machine learning. Nat. Biotechnol. 2022; 40:1520–1527. 35606422 10.1038/s41587-022-01307-0 PMC9546773

[30] Kodzius R. , Kojima M., Nishiyori H., Nakamura M., Fukuda S., Tagami M., Sasaki D., Imamura K., Kai C., Harbers M. et al. CAGE: cap analysis of gene expression. Nat. Methods. 2006; 3:211–222. 16489339 10.1038/nmeth0306-211

[31] Kwak H. , Fuda N.J., Core L.J., Lis J.T. Precise maps of RNA polymerase reveal how promoters direct initiation and pausing. Science. 2013; 339:950–953. 23430654 10.1126/science.1229386 PMC3974810

[32] Core L.J. , Martins A.L., Danko C.G., Waters C.T., Siepel A., Lis J.T. Analysis of nascent RNA identifies a unified architecture of initiation regions at mammalian promoters and enhancers. Nat. Genet. 2014; 46:1311–1320. 25383968 10.1038/ng.3142 PMC4254663

[33] Arnold C.D. , Gerlach D., Stelzer C., Boryn L.M., Rath M., Stark A. Genome-wide quantitative enhancer activity maps identified by STARR-seq. Science. 2013; 339:1074–1077. 23328393 10.1126/science.1232542

