# Supplementary material for: Defining the fine structure of promoter activity on a genome-wide scale with CISSECTOR
Source: Nucleic Acids Res. 2023 Apr 4;51(11):5499–511. doi: 10.1093/nar/gkad232 (PMC10287907; doi:10.1093/nar/gkad232)
Supplement: gkad232_Supplemental_File [file gkad232_supplemental_file.pdf]

Defining the fine structure of promoter activity on a genome-wide scale with CISSECTOR

Vincent D. FitzPatrick, Christ Leemans, Joris van Arensbergen, Bas van Steensel,
Harmen J. Bussemaker

SUPPLEMENTAL FIGURES

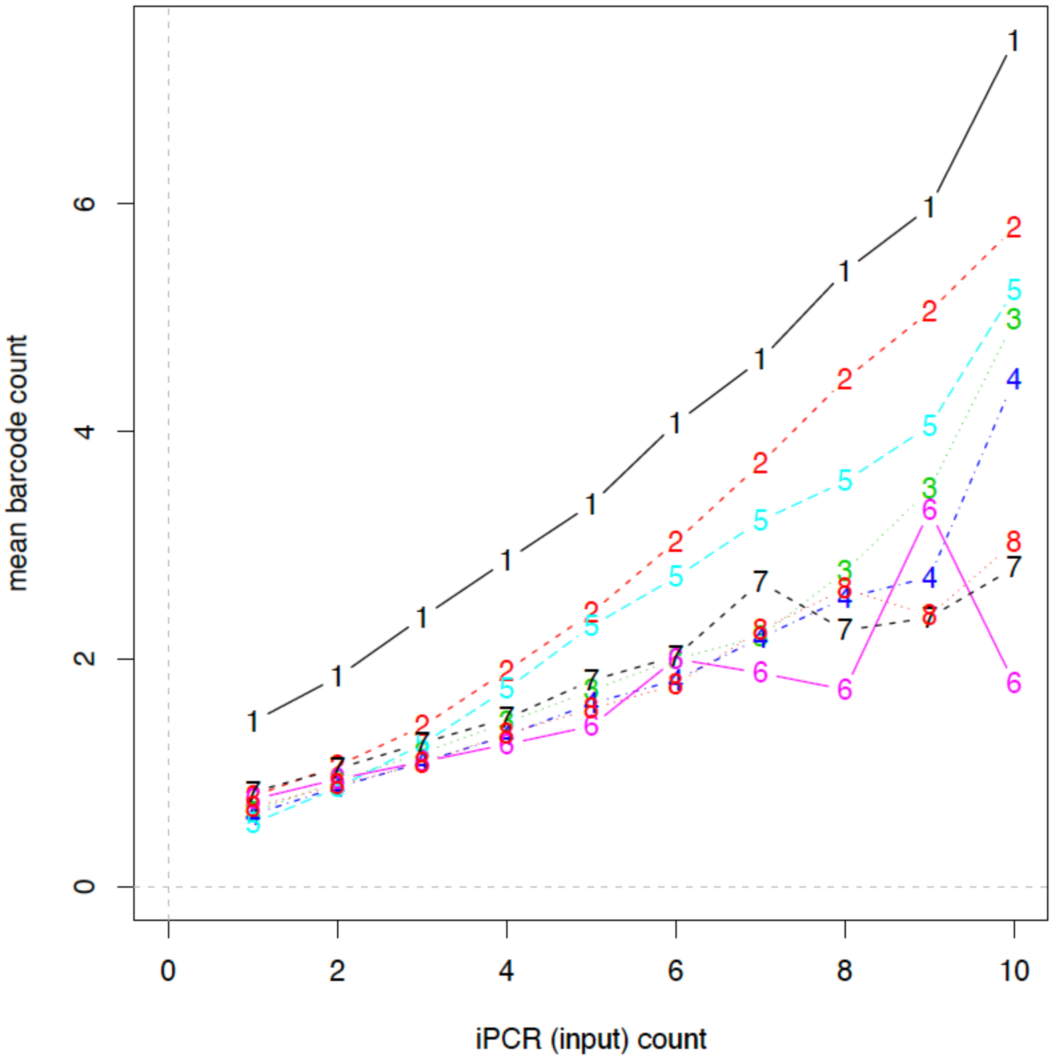

Supplemental Figure S1 – Library-specific mean expression shows a weakly non-linear relationship with input count. For each of the eight SuRE libraries used in the genome-wide GLM fits, the mean cDNA barcode count (*i.e.*, mean expression level) is shown for elements sharing the same inverted PCR (iPCR) count, for iPCR counts ≤ 10.

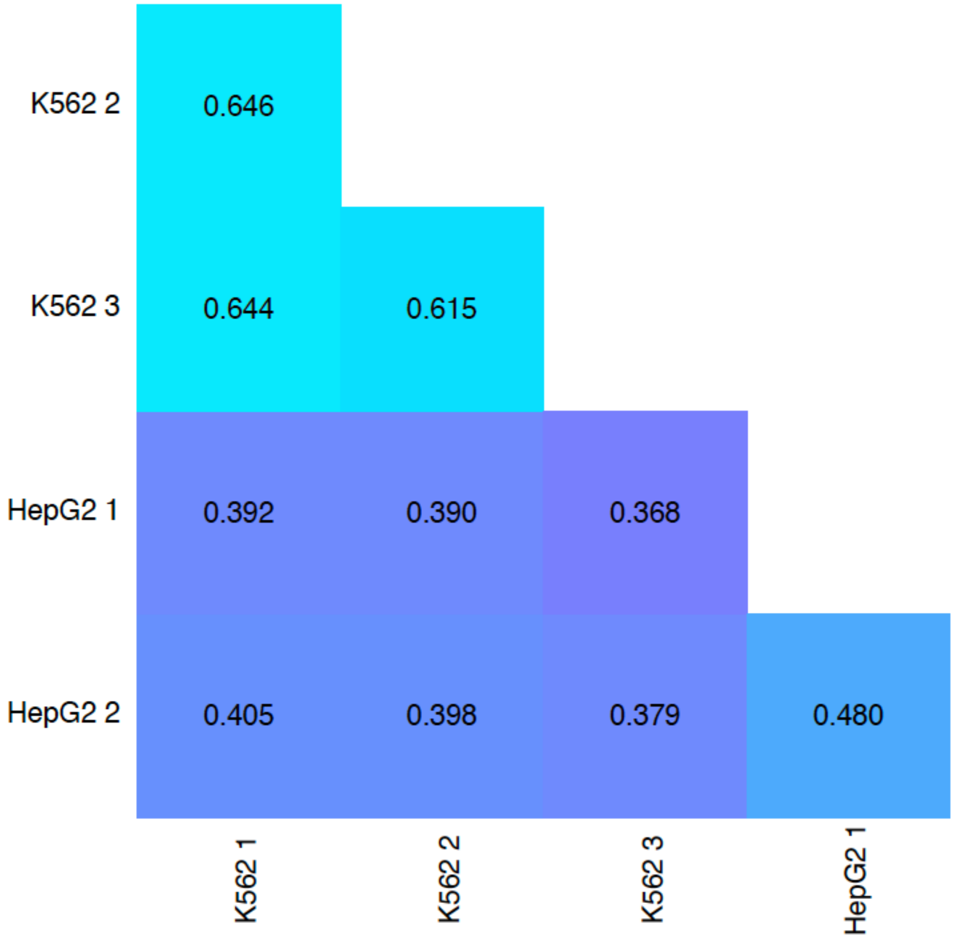

Supplemental Figure S2 – Pearson correlation matrix summarizing replicate-specific fits. To allow for a systematic comparison between intra-cell-type and inter-cell-type correlation, we performed separate fits for a subset of chromosome 17 using one cell type-specific replicate from each SuRE library. The Pearson correlation values shown reflect the similarity between the coefficient tracks for each pair of such CISSECTOR model fits.
